# More Than Skin-Deep: Integration of Skin-Based and Musculoskeletal Reference Frames in Localization of Touch

**DOI:** 10.1037/xhp0000562

**Published:** 2018-08-30

**Authors:** Renata Sadibolova, Luigi Tamè, Matthew R. Longo

**Affiliations:** 1 Department of Psychological Sciences, Birkbeck, University of London

**Keywords:** tactile localization, forearm, tactile spatial reference frame, body representation

## Abstract

The skin of the forearm is, in one sense, a flat 2-dimensional (2D) sheet, but in another sense approximately cylindrical, mirroring the 3-dimensional (3D) volumetric shape of the arm. The role of frames of reference based on the skin as a 2D sheet versus based on the musculoskeletal structure of the arm remains unclear. When we rotate the forearm from a pronated to a supinated posture, the skin on its surface is displaced. Thus, a marked location will slide with the skin across the underlying flesh, and the touch perceived at this location should follow this displacement if it is localized within a skin-based reference frame. We investigated, however, if the perceived tactile locations were also affected by the rearrangement in underlying musculoskeletal structure, that is, displaced medially and laterally on a pronated and supinated forearm, respectively. Participants pointed to perceived touches (Experiment 1), or marked them on a (3D) size-matched forearm on a computer screen (Experiment 2). The perceived locations were indeed displaced medially after forearm pronation in both response modalities. This misperception was reduced (Experiment 1), or absent altogether (Experiment 2) in the supinated posture when the actual stimulus grid moved laterally with the displaced skin. The grid was perceptually stretched at medial-lateral axis, and it was displaced distally, which suggest the influence of skin-based factors. Our study extends the tactile localization literature focused on the skin-based reference frame and on the effects of spatial positions of body parts by implicating the musculoskeletal factors in localization of touch on the body.

## Public Significance Statement

Tactile localization is conventionally studied in 2D space with respect to 2D somatotopic maps of the skin, and with reference to spatial locations of the 3D body parts in 3D space. Here we show that the touch is localized with reference to the skin in combination with a musculoskeletal arrangement of the limb underneath.

The skin is a two-dimensional (2D) sheet, stretched over the surface of the body. Somatotopic maps in the primary somatosensory cortex are also 2D, representing the skin as an orderly grid of overlapping receptive fields (RFs; Sur, Merzenich, & Kaas, 1980). The localization of touch has traditionally been conceived of as a process of linking a stimulus to a specific location on this 2D sheet (Head & Holmes, 1911; Longo, Azañón, & Haggard, 2010; Medina & Coslett, 2010; Rapp, Hendel, & Medina, 2002). Other research has investigated the process of “tactile spatial remapping,” by which information about location of touch on the skin is integrated with proprioceptive information about the location of body parts in space to perceive the location of a stimulus in three-dimensional (3D) external space (for a review on this topic see Heed & Azañón, 2014). However, there is a more basic way in which touch may be localized in 3D space, given that the skin surrounds the 3D musculoskeletal structure of the body. For example, the skin of the forearm is, in one sense, a flat 2D sheet, but in another sense is approximately cylindrical, mirroring the volumetric shape of the arm itself. This raises the question whether our experience of stimulus location is coded in a reference frame based on the skin itself, on the underlying musculoskeletal arrangement, or some combination of the two. In this study, we aimed to address this question.

At early cortical stages, tactile signals are processed within orderly somatotopic maps in primary somatosensory cortex (SI) wherein the spatial arrangement of neurons corresponds with the position of their receptive fields on body surface (Kaas, Nelson, Sur, Lin, & Merzenich, 1979; Penfield & Boldrey, 1937; Sereno & Huang, 2006). Indeed, stimulation of even single peripheral afferent fibers can elicit clear sensations localized to specific skin locations (Torebjörk, Vallbo, & Ochoa, 1987), suggesting that skin location is a basic property coded by afferent signals. Longo, Azañón, and Haggard (2010) recently introduced a model whereby the localization of touch initially takes place within early somatotopic maps and it is subsequently mapped onto a skin-centered body representation in higher brain regions. The model is consistent with localization performance of patients with left hemispheric damage (Rapp et al., 2002), which suggests a preserved somatotopy as manifested in accurate localization of touches with respect to one another, while showing the deficits in their overall mapping on the skin. The relative locations of perceived touches are preserved while there is an overall mislocalization in distal direction also by healthy participants for instance on the hand dorsum (Mancini, Longo, Iannetti, & Haggard, 2011; Margolis & Longo, 2015; Medina, Tamè, & Longo, 2018).

To determine the tactile locations on the 3D body, its posture and spatial locations of body parts must be factored in (Heed & Azañón, 2014). Sensory spatial information is represented in modality-specific reference frames to begin with. For instance, spatial location in the visual domain is computed from retinotopic map coordinates in a gaze-centered frame of reference (Crawford, Henriques, & Medendorp, 2011) while localization of touch on the skin is determined from locations on a somatotopic map and proprioception in body-centered, spatial reference frame (Heed, Backhaus, Röder, & Badde, 2016; Yamamoto & Kitazawa, 2001). A substantial amount of work has been done to study the integration of different spatial reference frames (Azañón, Longo, Soto-Faraco, & Haggard, 2010; Azañón & Soto-Faraco, 2008; Badde & Heed, 2016; Heed et al., 2016; Shore, Spry, & Spence, 2002). In the classic “crossed-hands” paradigm, for instance, the standard anatomical configuration with the right hand being to the right from the left hand changes, which impairs temporal order judgments for touches delivered on the hands (Badde, Heed, & Röder, 2016; Shore et al., 2002; Yamamoto & Kitazawa, 2001). The current theory for tactile localization on the body in any given posture in three-dimensional space posits a weighted integration of multiple types of spatial location codes which coexist in parallel for the optimal localization outcomes (Badde & Heed, 2016; Heed et al., 2016; Tamè, Wühle, Petri, Pavani, & Braun, 2017).

The above brief overview of two mainstream approaches in tactile localization research reveals an evident discontinuity between the research fields. Though the skin is a 2D sheet, it envelops the 2D volume of the body, whatever its posture in external space might be. Body segments are characteristically shaped by their musculoskeletal anatomy. Further, it is typical for movements and thus the underlying musculoskeletal spatial rearrangement to strain and displace the skin on body surface. For instance, a marked location at the *center* of the left posterior forearm in pronated posture will be displaced *laterally* when the forearm rotates into the supinated posture. Under the skin surface, the pronator muscles pull on the radius bone to cross it over the ulna, pivoting the hand until the thumb points medially toward the body. Conversely, the supinator muscle and biceps brachii pull the radius bone until it runs parallel with the ulna and the thumb points laterally away from the body (Gray, 1918/2000). As a consequence, the skin and superficial forearm muscles responsible for the elbow, wrist, and finger movements including the abduction, flexion, and extension, are also displaced in forearm rotation in a direction of the underlying bone and deep muscle movement.

To our knowledge, the musculoskeletal frame of reference has not been considered in the tactile localization literature. Nevertheless, it is not inconceivable that the mapping of touch in 2D space, given by a sheet of skin on body and its corresponding 2D-somatosensory representation, may be affected by the underlying anatomy of the 3D limb. Indeed, neurophysiological research with monkeys shows that arm movement direction and posture are represented in somatosensory cortex (Prud’homme, Cohen, & Kalaska, 1994; Sakata, Takaoka, Kawarasaki, & Shibutani, 1973). Further, the activity of tactile neurons was observed during arm movements without direct tactile stimulation (Cohen, Prud’homme, & Kalaska, 1994; Sakata et al., 1973). Moreover, the tactile signals produced by mechanoreceptors in skin on the hand dorsum convey movement-associated posture changes for the neighboring fingers (Edin, 1992; Edin & Abbs, 1991; Edin & Johansson, 1995). Furthermore, a recent study has shown that the response of somatosensory cortices following repetitive stimulation is mirrored in the motor cortex and that cortico-spinal excitability is modulated as a function of a temporal and spatial relationship between afferent stimuli (Tamè et al., 2015). Altogether, this evidence suggests an interactive relationship between the signals from skin, joints, and muscles in somatoperception.

Here we investigated the error of tactile localization in an explicit localization task. Participants were asked to point out the perceived locations of touches on their forearm while blindfolded (Experiment 1), and to mark them on a size-matched 3D image on a computer screen (Experiment 2). The aim of the study was to investigate the performance in a tactile localization task as a function of forearm torsion while the limb’s location in external space did not change. We hypothesized that if the localization judgments were made purely in skin-based reference frame, the perceived locations of tactile stimuli would follow the displacement of actual stimulus locations in each posture. In other words, the relative locations of perceived and actual touches would not differ as a function of forearm rotation. However, if the musculoskeletal factors play a role, the perceived tactile locations will show a systematic displacement from the actual touches consistent with a direction of muscular and skeletal rearrangement under the skin in each forearm posture. In other words, the mislocalization in medial and lateral direction would be expected respectively for the pronation and supination.

## Experiment 1

### Method

#### Participants

Twenty individuals participated in the study (12 females, 25.8 ± 7.8 years). All participants were predominantly right-handed, as assessed by Edinburgh handedness inventory (Oldfield, 1971; Mean ± *SD*: 93.6 ± 8.2). Written informed consent was obtained from all participants. The study was conducted in accordance with the Declaration of Helsinki, and was approved by the Psychology Ethics Committee, Birkbeck, University of London.

In related experiments, Pritchett and Harris (2011) investigated tactile localization error on the forearm as a function of a rotation of other body part (head), and Azañón, Radulova, Haggard, and Longo (2016) measured with temporal order judgments the tactile localization on forearm as a function of a change in forearm posture (Experiment 3). In a G*Power software (Faul, Erdfelder, Lang, & Buchner, 2007), the effect sizes from these studies (η_p_^2^ = 0.40, p. 231, and η_p_^2^ = 0.44, p. 1324, respectively) were converted to f(U) and used to calculate the sample size needed for a statistical power of 0.90 at alpha level 0.05. The power analysis revealed a sufficient sample size to be 19 and 17 participants, respectively. Thus, our sample of 20 should be appropriately powered to find an effect of similar magnitude.

#### Materials and procedure

**Figure 1 fig1:**
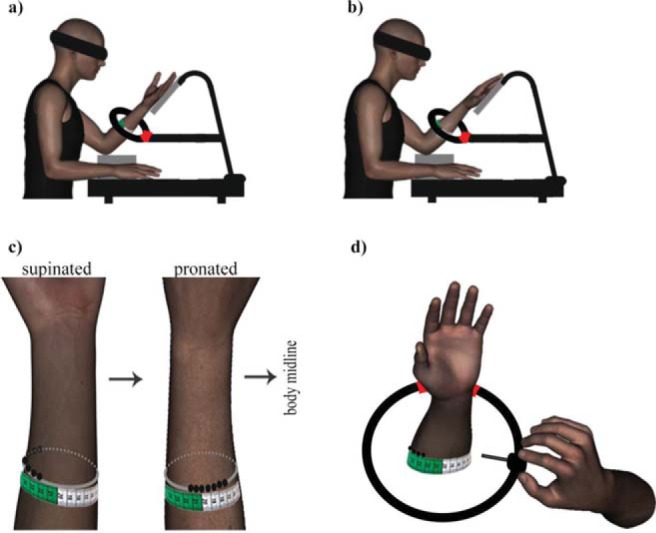
Experimental setup. Tactile localization judgments were made with the stimulated left forearm in supinated (panel a) or pronated (panel b) posture. On each trial, the participant was touched at one of seven locations which were marked 1 cm apart on their skin with a black pencil (black dots in panel c). The gray ellipse in panel c is used here for illustrative purposes to depict that the stimulus locations were positioned along the forearm’s circumference (i.e., they would form a straight line if the skin was flattened). Panel c additionally shows the displacement of the stimulus grid as a function of forearm rotation. The stimulus grid, drawn on the pronated forearm, would be displaced laterally with the displaced skin in forearm supination. While blindfolded, the participants moved a pointer along the ring around their left forearm, without making contact with the skin (panel d). The experimenter recorded the actual and perceived tactile locations on the tape measure which was attached to participant’s forearm at the level of the ring. There was a blind spot (marked in red) where the ring was attached to its holder.

Figure 1 shows the experimental setup. The participant sat at a table. Their elbow rested on a soft cushion and their hand rested on a tilted wooden platform 25 cm above the table, resulting in the forearm forming approximately 90° angle with the upper arm. In the *supinated posture* (Figure 1a), the hand dorsum rested on the platform, with the palm facing up. In the *pronated posture* (Figure 1b), in contrast, the palm rested on the platform, with the dorsum facing up. The angle of the forearm and that of the platform was approximately 60° relative to the table.[Fig-anchor fig1]

A piece of rubber tubing (outer diameter = 2 cm) was used to form a ring of approximately 27 cm in diameter. The ends of the tube were glued together. The ring was attached to the structure at the approximate forearm’s midlength level. Additional elbow support could be added for participants with shorter forearms. The position of the participant’s forearm was adjusted to be aligned at the center of the ring, without actually contacting the tubing. Thus, the ring formed a circle around the participant’s forearm separated by ∼9-cm to 10-cm distance from the skin. There was a disk with a pointer which could easily be moved along the ring (Figure 1d) with the exception of the farthest location where the ring was attached to the structure (∼30°). The pointer was 5-cm long and it pointed toward the center of the circle toward the forearm regardless of the disk location on the ring (Figure 1d). The tube material was firm to prevent the ring deformation by a pressure that the participant might apply against it when moving the pointer.

Prior the experiment, the participant saw the structure and their forearm inside at the center of the ring. They were allowed to move the pointer with their right hand to experience it pointing to different locations along their forearm’s circumference. They were then blindfolded. A flexible plastic tape measure was placed on their skin at forearm’s midlength running along the forearm’s circumference (see Figure 1). A paper clip was used to hold the tape measure ends together at the most distal point, which was on the anterior side of the pronated forearm. Using a black pencil, the experimenter drew seven points 1-cm apart immediately next to the tape measure attached to the skin (Figure 1c). Relative to the center of the pronated forearm, two points were marked to the participant’s left and four points were marked toward their body midline. Together with the central point, there were seven stimulus locations in medial-lateral direction. The points labeled from 1 to 7 and the distance on the tape measure increased in a direction toward participant’s body midline, that is, in anticlockwise direction along the forearm’s circumference.

The participant’s task was to use a pointer to indicate the location of tactile stimuli along the circumference of the forearm. Localization along the forearm’s proximo-distal axis was not assessed in Experiment 1 due to a fixed location of the pointer on this axis. A script written in MATLAB (Mathworks, Natick, MA) was used to run the experiment. There were two starting locations of the pointer, one at each side of the blind spot where the tube was attached to its holder structure. Each trial started with an instruction for the experimenter about the pointer position and tactile stimulus locations. The participant’s head was oriented toward their left forearm. On each trial, the experimenter applied a tactile stimulus using a Von Frey filament (60 g force) at one of the seven marked locations. The stimulations lasted ∼1 s each. The experimenter then guided the participant’s right hand toward the pointer. The participant moved the pointer along the ring to indicate the perceived location of the touch. The experimenter then recorded the actual and perceived locations as corresponding locations on the tape measure on the participant’s skin.

The key manipulation was the forearm posture created by its torsion, the *pronated forearm* condition and the *supinated forearm* condition. There is a notable skin displacement in the clockwise direction along the forearm’s circumference in the latter condition (Figure 1c). As a result, the marked stimulus locations move to the lateral side of the forearm, thus becoming approximately aligned with the lateral position of the thumb. In contrast, the central stimulus location in the pronated posture is at almost 90° angle in the clockwise direction relative to the medial side of the forearm. There were two blocks for each forearm posture counterbalanced across participants using a Latin square randomization. During trials, the participant was instructed not to move their left forearm. There were 28 trials in each block with individual stimulus locations stimulated four times each. Their order was randomized.

#### Data analysis

For each trial, the actual and perceived locations of the touch were recorded in centimeters on the tape measure running anticlockwise around the circumference of the participant’s forearm. We assessed the ability of participants to localize the touch on their forearm by computing two different classes of localization error. The first class (constant error) quantifies systematic displacement of the perceived touch relative to its actual location. It was calculated for each stimulated point in each forearm posture as a difference of the average of localization attempts and their corresponding actual location. Thus, positive values represent mislocalization of touch in the anticlockwise direction, while negative values represent mislocalization in the clockwise direction. The aim of the analysis was to quantify the direction and magnitude of the displacement of perceived touches from their actual locations. The second class or localization error is associated with the precision and it was computed as a variability (standard deviation) of individual localization attempts.

For each localization error type, we first report an overall error in one-sample *t* test comparisons for each posture. An ANOVA follows, with the posture and actual stimulus locations as independent variables. We treated the locations as a categorical variable (seven levels) in ANOVA. However, in the post hoc tests, we assessed the trends across the locations by fitting the linear regression model to the data. This approach allowed for a comprehensive study of the effects which would not be detected if the linear regression was fitted by default to all data. However, the linear regression in the post hoc tests was a viable alternative to multiple *t* test comparisons which would increase the complexity of the results.

### Results and Discussion

**Figure 2 fig2:**
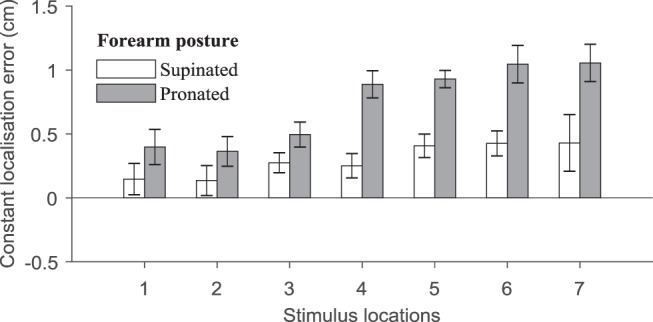
The constant localization error along the forearm’s circumference at the forearm’s midlength. The positive values represent the displacement of the perceived touch relative to its actual location in anticlockwise direction. The larger values on the *x*-axis are for more medial locations of the stimulus array. The error bars are within-subject standard error (Loftus & Masson, 1994).

Figure 2 shows a bias to perceive tactile stimuli as being farther anticlockwise than they actually were, both in the supinated posture (*M*: 0.30 cm, *SD*: 0.53), *t*(19) = 2.51, *p* = .02, *d* = 0.56, and the pronated posture (*M*: 0.74, *SD*: 0.48), *t*(19) = 6.84, *p* < .0001, *d* = 1.53. The constant localization error was submitted to a repeated-measures analysis of variance (ANOVA) with the forearm posture (supinated and pronated) and the actual stimulus location (seven levels) as independent variables. The anticlockwise displacement of the perceived touch was larger on the pronated forearm than in the supinated posture, *F*(1, 19) = 11.05, *p* < .01, η_p_^2^ = 0.37, suggesting a greater perceptual “pull” toward the medial side in this posture. Additionally, we found a trend for the main effect of grid locations, *F*(1.83,34.70) = 3.38, *p* = .05, η_p_^2^ = 0.15 (Greenhouse-Geisser corrected; GG-corr) which was driven by an interaction (see Figure 2), *F*(6, 114) = 2.42, *p* = .03, η_p_^2^ = 0.11. To determine the interaction, we used least-squares regression for each participant to fit a linear model to the data across stimulus locations, and we assessed the slope coefficients across the forearm postures in the second-level analysis. On the pronated forearm, the mislocalization increased in magnitude for grid locations closer to the medial side (*M*: 0.13 cm/location, *SD*: 0.21), *t*(19) = 2.88, *p* = .02, *d* = 0.64. No such increase was observed for the supinated posture (*M*: 0.06, *SD*: 0.21), *t*(19) = 1.21, *p* = .24, *d* = 0.27 (Holm-Bonferroni corrected; HB-corr). In other words, we found a perceptually stretched grid on the pronated forearm but not on the supinated forearm.[Fig-anchor fig2]

Next, the standard deviation of individual localization attempts was submitted to a repeated measures ANOVA with the forearm posture (supinated and pronated) and the actual stimulus location (seven levels) as independent factors. There were no differences in localization variability in the supinated (*M*: 0.94, *SD*: 0.25) and pronated (*M*: 0.89, *SD*: 0.23) postures, *F*(1, 19) = 0.65, *p* = .43, η_p_^2^ = 0.03, or between the individual stimulus locations, *F*(6, 114) = 0.99, *p* = .44, η_p_^2^ = 0.05, nor was there an interaction effect, *F*(4.31,81.97) = 1.18, *p* = .33, η_p_^2^ = 0.06 (GG-corr).

In conclusion, the magnitude of constant localization error is relatively small (<1 cm) and the response variability is low (standard deviation <1 cm). The touch was mislocalized in medial direction toward body midline in the pronated posture. However, it was also mislocalized toward the body, away from the lateral side and thus contrary to what was predicted for the supinated posture. In Experiment 2, we explored this finding as a possible movement confound. Given the anatomical constraints of the body, the right-hand movement in localization of leftmost touches on a supinated forearm becomes more effortful. Because the perceived stimulus locations felt further apart on the pronated forearm, it is plausible that the same perceptual stretch of the stimulus grid failed to be observed with the increased movement difficulty in the supinated posture. A stretch reduction for the leftmost half of the grid on the supinated forearm would inadvertently result in an apparent localization bias toward the body when there really is none. To investigate this possibility, we conducted the second experiment using a different response modality whereby participants indicated the perceived tactile locations on a 3D image of the forearm. We additionally expanded the experiment by including a 2D stimulation grid.

## Experiment 2

While the emphasis in the previous experiment was on localization in the medial-lateral axis, one of the aims of Experiment 2 was to expand this investigation by including the proximo-distal dimension. Thus, we would study the tactile localization in 3D space given by the width and length of a *curved* stimulation grid. In Experiment 2 we eliminated the motor feedback of the contralateral hand and we ameliorated the potentially confounding variability in response difficulty across the grid locations. Further, the tube around the forearm might be criticized for being used as a magnified approximation of forearm circumference and as such to cause the perceptual stretch of the grid. To address these concerns, and to study tactile mislocalization with the added grid dimension, we conducted the Experiment 2. While previous studies used 2D body part silhouettes on a computer screen to mark the perceived tactile locations (Mancini et al., 2011; Margolis & Longo, 2015; Sadibolova, Tamè, Walsh, & Longo, 2018), we adapted this paradigm for a study of tactile localization on 3D forearm. The stimuli and the data from both experiments (Sadibolova, Tamè, & Longo, 2018) are available in online supplemental material.

### Method

#### Participants

An independent sample of 20 participants was recruited (14 females, 27.1 ± 10.7 years). They were predominantly right-handed, as assessed by Edinburgh handedness inventory (Oldfield, 1971; Mean ± *SD*: 87.6 ± 17.5). Written informed consent was obtained from all participants, and the experiment was approved by the local ethical committee and was consistent with the principles of the Declaration of Helsinki.

#### Materials and procedure

**Figure 3 fig3:**
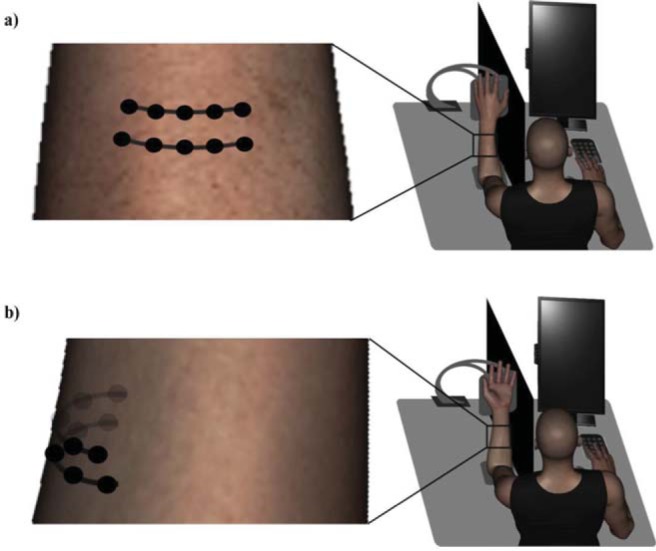
Experimental setup. Tactile localization judgments were made with the stimulated left forearm in pronated (panel a) or supinated (panel b) posture. The stimulus locations (black dots), including those on the unseen side of the forearm (gray transparent dots in panel b), are arranged in 2 × 5 grid as shown in the zoomed-in images on the left side. The grid was drawn on a pronated forearm, and it moved laterally with the displaced skin in forearm supination. If the skin was flattened up, the grid locations would form a rectangle (pronated posture) or a horizontally aligned rhombus (supinated posture). The grid is skewed on a supinated forearm due to slightly larger displacement of the top row (see also Figure 4). After each stimulation, a size-matched image of the forearm and a white cross at its center appeared on participant’s monitor. By pressing keys on a number pad, the participant proceeded through the images of a forearm at progressively changing view angles along its circumference, which gave an impression of viewing the rotating three-dimensional forearm (see also Figure 5). The participant selected the view angle and moved the cross on the vertical axis to mark the perceived tactile locations.

The participant was seated at the table. Their task was to mark on a 3D forearm on a computer screen the locations corresponding with those of the perceived touches on their actual forearm. The view of the stimulated left forearm with the elbow resting on a soft cushion on the table was occluded by a black foamboard sheet. The hand rested on an elevated platform used in Experiment 1. The ring was removed from the structure. The forearm position was identical to that described in Experiment 1. The participants faced a 22 in. × 14 in. monitor (tilted at 90°) and they responded with their right hand using a number pad (see Figure 3). The view of their right hand and forearm was also prevented by an occluder. The experiment was run with a script written in MATLAB using PsychToolbox (Brainard, 1997; Pelli, 1997).[Fig-anchor fig3]

The experimenter marked with a black pencil a crease at the participant’s left elbow and a line around the wrist–hand intersection in the *pronated forearm* posture. Their distance was recorded with a tape measure as the length of the forearm. The width of the forearm was taken at its center-point with a caliper given the round surface. To record the width, another sheet of a foamboard was temporarily placed under the forearm. The caliper ends were extended by approximately 3.5 cm each to reach the foamboard for the measurement at a constant angle. The medial-lateral center at this level was marked as the center of the forearm to enable drawing of the stimulus grid locations on the skin and for their later alignment with the perceived locations of touch marked on an image of the forearm. To draw the grid, the experimenter drew a straight line from the center of the wrist passing through the forearm center. Two horizontal rows of the grid, 0.5 cm in proximal and distal direction from the forearm center, were perpendicular to this line. There were five stimulation points 1-cm apart in each row with the central points on the line along the forearm. We used flexible plastic right-angle rulers to mark these locations.

**Figure 4 fig4:**
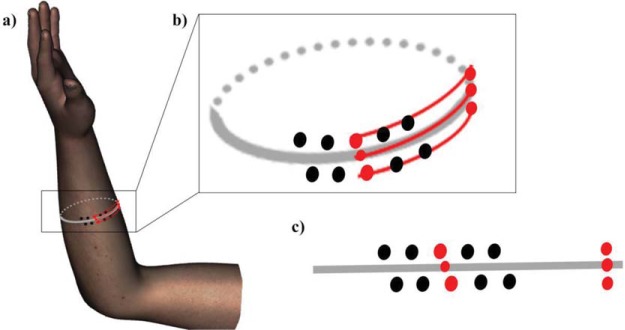
Skew of the grid in the supinated posture. The center of the supinated forearm was marked as per procedure used for the grid-drawing on the pronated forearm. In all three panels, it is shown as the middle of the rightmost three red dots. These three points (0.5 cm apart on a proximo-distal axis) were *not* used for stimulation. Panels a–b show the laterally displaced 2 × 5 stimulus grid on a supinated forearm which was aligned with forearm’s medial-lateral axis (gray ellipse). If the skin was flattened, the stimulus locations would form a rhombus (panel c). The skew was due to a slightly larger displacement of the top row in supination (∼0.3 cm). We recorded the distances (red lines in panels a–b) between the grid’s central points and each of the three reference points. With the forearm circumference approximated to a circle with a known radius for each participant, these measurements were then used in the data analysis stage to determine the relative locations of the actual and perceived touches.

As shown in Figures 3 and 4, the grid moves with the skin in clockwise direction along forearm’s circumference, and it is skewed when the *dorsum* of the hand rests on the platform (*supinated forearm* condition). The skew was caused by differences in clockwise displacement across rows, the magnitude of which is slightly larger for the upper row closer to the wrist (mean: 0.31 cm, *SD*: 0.06). The overall grid displacement between the postures is consistent in both experiments (Figure 1c and 3). For the data analysis purposes, a new forearm center and central points of each row were marked on the *supinated* forearm following the procedure described earlier. A rubber band was placed around its circumference in order not to deviate from the forearm midlength level. The tactile stimuli would not be applied to these three extra points. Their distance from the corresponding points of the grid in supinated posture was used to calculate the shift and skew of the grid in data analysis stage (see Figure 4). With the forearm circumference approximated to a circle, it was straightforward to quantify the skew both in centimeters and angle degrees, and compute the tactile mislocalization using these measurements.[Fig-anchor fig4]

**Figure 5 fig5:**
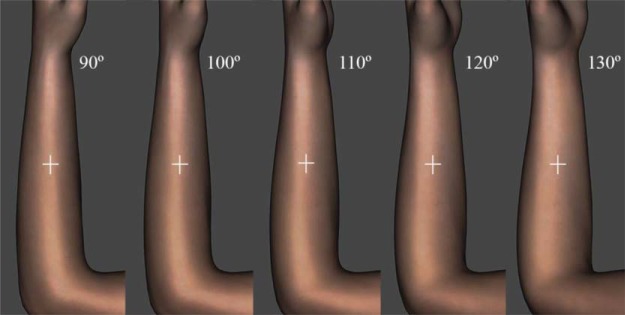
Three-dimensional forearm. The figure shows five of 360 snapshots of a forearm from different angles of the view in steps of 10°. A single image would appear on a participant’s screen with a white cross at the forearm’s center. The participant could move the cross in vertical direction but not horizontally. They could however change the angle of rotation which would result in a white cross moving along the forearm’s circumference.

The forearm circumference at its midlength is approximately circular with a radius determined from the participant’s forearm width. It was thus straightforward to quantify the individual grid locations in angles and in centimeters on the medial-lateral axis. The participants marked perceived locations of touches at corresponding locations on an image of a forearm on a computer screen. To prevent the 2D compression of the curved surface, images of a generic three-dimensional forearm in DAZ Studio (DAZ 3D, Salt Lake City, Utah) were taken from all angles of a view in anticlockwise direction at 1° increments (360 images; cf. Figure 5). The pronated forearm’s center was used as a zero angle for both, the actual forearm and the forearm image. This arrangement later allowed the calculation of medial-lateral displacement of perceived touch from its corresponding actual location.[Fig-anchor fig5]

The images were cut to include a part of the hand with sufficient clues about the position of the palm, dorsum, thumb, and the ulnar edge of the hand. The upper arm was invisible and thus participants viewed the forearm only in its full length including the elbow. The center of the forearm at 0° angle on the image, which corresponded with that on the participant’s pronated forearm, was at the center of the image. Thus, we were able to change the image length and width for each participant to match in size their actual limb and the forearm on the screen while maintaining the center of the forearm at the center of the image.

A single image would appear on a screen with a white cross at the forearm center. The participants were asked to mark the perceived location of the touch with a cross at the corresponding location on the image. The cross on the image at 0° angle was at the location corresponding with that of the grid center on the actual pronated forearm. The participants were unable to move the cross in medial-lateral direction and thus it always appeared at forearm’s medial-lateral center. They were however able to rotate the view using the “1” and “3” keys on the number pad. Pressing the “1” key rotated the forearm in the anticlockwise direction, while pressing the “3” key rotated the forearm in the clockwise direction. Participants were able to move the cross on the vertical axis using the “0” (downward) and “5” (upward) keys. The “1” and “3” keys pressed on their own resulted in a slow rotation in 1° steps. When pressed simultaneously with “enter” the change was faster in 10° steps. Similarly, the cross could be moved at a slower rate of 0.1 cm when the “0” and “5” keys were pressed alone, and at 2-cm rate of change when either of these keys was pressed simultaneously with “enter.” The participants were encouraged to move the cross to the proximity of their response location faster for the memory trace not to deteriorate and then to use the slow adjustments to mark the perceived location accurately.

The individual trials were presented in four blocks of 40 trials each. There were two blocks for each forearm posture counterbalanced across participants using a Latin square randomization. The experimenter would apply the touch with Von Frey filaments (60 g) at one of 10 stimulus locations (2 × 5 grid) and press the key for the forearm image to appear on the participant’s screen. The first image would be of a forearm at one of the 0°, 90°, 180°, or 270° view-angles. The vertical position of the response cross was always at the longitudinal center of the forearm. The trial order was randomized.

#### Data analysis

Care was taken for the center of the pronated forearm to overlap with that of the pronated forearm on the image (0° view angle). This was the center of the actual stimulus grid on the pronated forearm. The grid center and its individual locations for the supinated forearm posture were calculated as a distance in centimeters relative to this starting position using the grid displacement measurement recorded for each participant. The grid center in each posture was used as an origin of the coordinate system for the computation of perceived and actual touch locations. Its ordinate ran along the proximo-distal axis while the abscissa ran along the circumference of the forearm. While the raw actual stimulus locations were in centimeters relative to the grid center, the raw perceived locations had to be converted from angles in degrees on the *x*-axis and pixels on the *y*-axis. On a forearm circumference approximated to a circle, the angle of each response and the forearm width were used to compute the arc length, that is, the distance in centimeters relative to the grid center.

The resulting *x* and *y* coordinates of each actual and perceived tactile location were processed in a manner identical to Experiment 1. The constant localization error was calculated for each stimulated point in each forearm posture as a difference of averaged localization attempts and their corresponding actual location. The positive and negative values at the *x*-axis represented the localization error along the forearm’s circumference, respectively, in anticlockwise and clockwise direction. The distal and proximal displacement was represented by positive and negative values on the *y*-axis, respectively. The variable localization error was calculated as a standard deviation of individual localization attempts for each stimulated point in each posture.

For each localization error type, we first report an overall error in one-sample *t* test comparisons for each posture. An ANOVA follows, with the posture (two), stimulus grid rows (two), and stimulus grid columns (five) factors. As in Experiment 1, the grid locations are treated as a categorical variable in ANOVA. In post hoc tests, however, the trends across locations were assessed by fitting a linear regression model to the data.

### Results and Discussion

**Figure 6 fig6:**
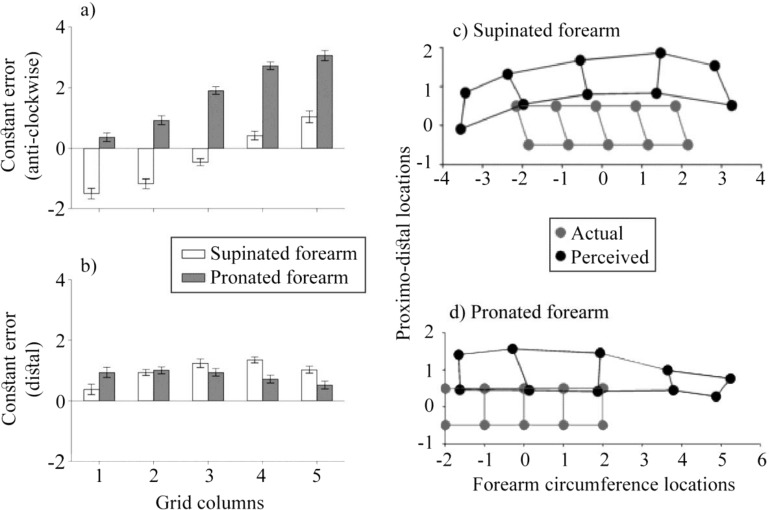
The constant localization error. Panels a–b show the anticlockwise and distal mislocalization of perceived touches relative to their actual locations (in centimeters) for individual grid columns collapsed across grid rows. The error bars are within-subject standard error (Loftus & Masson, 1994). The relative position of actual and perceived grid locations is shown in panels c–d. The perceived locations are displaced anticlockwise on the pronated forearm but not on the supinated forearm. A distal displacement and a perceived stretch of the grid along forearm’s circumference is observed for both postures.

The top left panel of Figure 6a shows constant localization bias at the medio-lateral axis. As in Experiment 1, the perceived locations of tactile stimuli were displaced medially in anticlockwise direction on the pronated forearm (*M*: 1.79 cm, *SD*: 0.95), *t*(19) = 8.48, *p* < .0001, *d* = 1.89. However, unlike in Experiment 1, there was no overall anticlockwise localization bias in the supinated posture (*M*: −0.33, *SD*: 1.46), *t*(19) = 1.02, *p* = .32, *d* = 0.23 (Holm-Bonferroni corrected *p* values; HB-corr). Thus, using a different response modality, we replicated tactile mislocalization in medial direction consistent with the underlying skeletal and muscular rearrangement in forearm pronation. However, we found no such bias for the supinated forearm having eliminated the increased movement difficulty for the leftmost grid locations which were indeed the concern in Experiment 1.[Fig-anchor fig6]

Constant localization error in medial-lateral orientation was further assessed in a repeated-measure ANOVA with forearm posture (supinated and pronated), grid rows (two levels), and grid columns (five levels) as independent variables. We replicated the larger anticlockwise localization error for the pronated forearm (Figure 6a and 6d), *F*(1, 19) = 32.36, *p* < .001, η_p_^2^ = 0.63. On the whole, the mislocalization increased in magnitude for more medial grid columns, *F*(2.10,39.93) = 66.27, *p* < .001, η_p_^2^ = 0.78, for both rows of the grid, *F*(4, 76) = 2.10, *p* = .09, η_p_^2^ = 0.10, and both postures, *F*(2.89,54.99) = 1.05, *p* = .38, η_p_^2^ = 0.05. The post hoc *t* tests confirmed increasing slopes across grid columns for both the pronated and supinated forearm (*M*: 0.72 cm, *SD*: 0.32), *t*(19) = 10.15, *p* < .001, d_z_ = 2.27, and (*M*: 0.67, *SD*: 0.36), *t*(19) = 8.24, *p* < .001, d_z_ = 1.84, respectively (HB-corr). Thus, using a different response modality, we also replicated a perceptual stretch of the grid in the pronated posture. This rules out as a causal factor potentially magnified forearm circumference due to a large ring surround in the Experiment 1. Moreover, we found a similar stretch for the supinated forearm which was not observed in Experiment 1 due to aforementioned movement complication. This stretch is shown in Figure 6c and as an increasing anticlockwise localization bias for supinated forearm in Figure 6a. To summarize, the evidence shows a perceptual stretch of the grid in both postures. However, only the grid on the pronated forearm was displaced. For both response modalities, the touch was in this posture mislocalized medially which is consistent with the direction of rearrangement in underlying skeletal and muscular structure and a corresponding change in hand posture. In contrast, the grid was not moved in supinated posture, given that it was already aligned at the lateral side. In other words, with the actual grid centered at the most lateral location in forearm supination, the perceptual decentering and thus overall clockwise or anticlockwise displacement of the grid in this posture would be in medial direction. Thus, no decentering is consistent with our prediction. It should be noted that the forearm rotation is limited, that is, the forearm does not rotate by >180 degrees which would account for why the grid was not perceptually shifted further clockwise.

Figure 6b shows constant localization error in proximo-distal axis. The participants judged tactile stimuli to be closer to the wrist on both the supinated and pronated forearm (*M*: 0.82, *SD*: 1.60), *t*(19) = 2.31, *p* = .04, *d* = 0.52, and (*M*: 0.98, *SD*: 1.71), *t*(19) = 2.58, *p* = .04, *d* = 0.58, respectively (HB-corr). This mislocalization was similar across the postures, *F*(1, 19) = 1.75, *p* = .20, η_p_^2^ = 0.08, and it did not differ across grid rows on the whole, *F*(1, 19) = 1.55, *p* = .23, η_p_^2^ = 0.08, or across the rows in individual postures, *F*(1, 19) = 0.65, *p* = .43, η_p_^2^ = 0.03. Thus, there was no perceptual stretch of the grid at the proximo-distal axis. There was, however, a main effect of grid column, *F*(2.57, 48.74) = 3.26, *p* = .04, η_p_^2^ = 0.15, which was modulated by an interaction with the posture, *F*(2.69, 51.05) = 5.18, *p* = .01, η_p_^2^ = 0.21 (GG-corr). Figure 6b suggests the interaction to be driven by differences across postures in the reduced distal error at the grid ends. The distal bias was smaller at the location 1 on supinated forearm, *t*(19) = 2.59, *p* = .04, d_z_ = 0.58, while it was reduced for pronated forearm at Locations 4 and 5, *t*(19) = 3.44, *p* = .01, d_z_ = 0.77 and *t*(19) = 2.50, *p* = .04, d_z_ = 0.56, respectively (HB-corr). There was no three-way interaction, *F*(2.73, 51.74) = 2.03, *p* = .13, η_p_^2^ = 0.10 (GG-corr).

**Figure 7 fig7:**
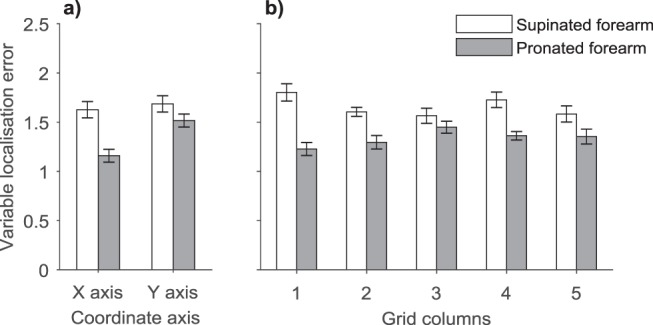
The variability in localization error. Panels a shows the variability in localization attempts for each forearm posture at the *x* and *y*-coordinate axes (along forearm’s circumference and in proximo-distal direction), respectively. Panel b shows the response variability across postures at individual grid columns. The error bars are within-subject standard error (Loftus & Masson, 1994).

Finally, we conducted ANOVA with the response variability at each axis, forearm posture (supinated and pronated), grid rows (two levels), and grid columns (five levels) as independent variables. Unlike in Experiment 1, the variable error was smaller on the pronated forearm, *F*(1, 19) = 14.41, *p* = .001, η_p_^2^ = 0.43, and it differed across postures and coordinate axes (see Figure 7), *F*(1, 19) = 7.50, *p* = .01, η_p_^2^ = 0.28. The post hoc tests showed that there was less response variability in medial-lateral orientation than on proximo-distal axis on the pronated forearm, *t*(19) = 2.72, *p* = .03, d_z_ = 0.61, but not on the supinated forearm, *t*(19) = 0.36, *p* = .72, d_z_ = 0.08. The reduced precision of localization in proximo-distal axis is consistent with the literature (Cody, Garside, Lloyd, & Poliakoff, 2008). The lack thereof for the supinated forearm may be related to a possibly larger skin stretch in this posture (Cody, Idrees, Spilioti, & Poliakoff, 2010). This would also explain the response variability being generally larger for the supinated forearm as reported earlier. Additionally, there was an interaction between the posture and grid columns (see Figure 7), *F*(4, 76) = 2.68, *p* = .04, η_p_^2^ = 0.12, which was not modulated by grid rows, *F*(2.40,45.59) = 0.25, *p* = .82, η_p_^2^ = 0.01 (GG-corr), or coordinate axes, *F*(4, 76) = 1.29, *p* = .28, η_p_^2^ = 0.06. The post hoc tests showed that the response variability was larger in supinated posture with the grid at the lateral side of the body for all grid columns (*p* < .001) except the central column 3 and 5 (*p* > .11). All other main effects and interactions were nonsignificant (*p* > .14).[Fig-anchor fig7]

## General Discussion

We reported two experiments investigating the influence of reference frames based on the skin and musculoskeletal structure in localization of touch on the left forearm. If localization is based entirely on a skin-based reference frame, the localization judgments should have followed the actual stimulus locations displaced with the skin displacement in forearm rotation, and have been similar for the pronated and supinated forearm postures. In contrast, if tactile location is also referenced to a frame based on the overall musculoskeletal structure underneath the skin, systematic biases in a direction of skeletal and muscular movement in arm rotation should be observed. Specifically, in medial direction on a pronated forearm and lateral direction on the supinated forearm. We report that the touch at *the same* skin location was indeed mislocalized in a different magnitude and direction as a function of forearm rotation. While the perceived touches were displaced medially relative to their actual locations on a pronated forearm, this displacement was either significantly reduced (Experiment 1), or absent altogether (Experiment 2) on a supinated forearm.

The mislocalization toward the medial side of the forearm was greater in the pronated posture than on the supinated forearm, irrespective of the response modality. Further, the mislocalization on the pronated forearm in both experiments was such that in addition to being displaced toward the medial side, the perceived locations were also farther apart along the forearm’s circumference than in the actual stimulus grid. Nevertheless, the evidence was mixed for the supinated forearm when the actual grid locations moved laterally with the displaced skin. Whereas the right locations of the grid were perceived to be farther right relative to their actual position, the left grid locations either show the mislocalization in the opposite, left direction (Experiment 2), or no displacement at all (Experiment 1). Thus, the grid was perceptually stretched and centered on forearm’s lateral side in Experiment 2, while this stretch was reduced for the left half of the grid in Experiment 1, causing thus an apparent decentering toward the torso.

The left half of the grid on supinated forearm showing the differences in stretch across response modalities is on the side of the forearm which is not seen from the egocentric perspective. In Experiment 2, the relative position of the limb to torso was not explicit (i.e., not shown on a computer screen) while the forearm could have been rotated 360° around the medial-lateral axis. One possibility could be that the perceived locations in Experiment 1 felt closer to the body center, that is, the torso, but the responses on the images in Experiment 2 would not reflect this bias. This interpretation would be in line with the theory of coexisting parallel spatial reference frames being weighted differently depending on task demands (Badde & Heed, 2016). However, a more likely interpretation concerns a confounding factor of contralateral hand movements which was eliminated in Experiment 2. Although the participants *could* move the pointer up to 60° clockwise from the position of the thumb in the supinated posture, the righthand movement became more effortful in this direction and care had to be taken not to touch the left forearm. This would have inadvertently caused the differences between two response modalities in the stretch of the left side of the grid across two experiments. We thus consider the evidence for the torso-centered mislocalization for the supinated forearm in Experiment 1 to be unreliable, as it may result from a potentially confounding effect of anatomical movement constraints.

The stretch patterns observed for both postures in both response modalities further eliminate the possibility of a general mislocalization toward the body midline. The error in the supinated posture increases at more medial locations of the grid compared with the grid center which is on the forearm farthest laterally. This seemingly suggests a perceptual “pull” toward the body midline which increases progressively with stimulus proximity to the body midline. However, there was *no* mislocalization at the leftmost end of the grid on the pronated forearm which is closer to the body midline than all grid locations in the supinated posture. This argues against a general tendency to mislocalize the touch toward the body midline.

Our evidence argues against the localization of touch based solely on a skin-based reference frame. Instead, it suggests that the musculoskeletal reference frame is likely to play a role in tactile localization too. The mislocalization differs between postures in a manner consistent with a direction of a change in muscular and skeletal arrangement driving the forearm rotation and pivoting the hand at 180° angle. Thus, the stimulus grid already positioned laterally on the forearm is not perceptually displaced from its location whereas the grid misaligned relative to the medial side is perceptually pulled toward it. The neighboring hand posture may be a contributing factor, the influence of which we attempted to reduce by positioning the grid farther away from wrist at the forearm’s midlength, and by ensuring that the hand was not seen at all (Experiment 1), or seen only partly on response images in the visual task (Experiment 2). Moreover, it would be interesting to investigate what is a “default” or “natural” forearm posture in a prototype body representation (Romano, Marini, & Maravita, 2017). This would be a natural next step to address in future experiments. Thereof, should one of the postures be “preferred” to the other, it would be interesting to explore in different scenarios the performance accuracy across two postures.

The skin-based influences were also observed. The medial-lateral stretch is consistent with the distortion of skin-based perceptual maps on the dorsum of the hand attributed to oval-shaped receptive fields of somatosensory neurons on the hairy skin of the arm (Longo & Golubova, 2017; Longo & Haggard, 2011). However, the skin stretch due to forearm rotation should also be considered. When skin is stretched, the receptive fields of touch receptors are likely to become larger and their spacing widens. This could explain the perceptual stretch of the grid, that is, perceiving its relative locations further apart. It should be noted, however, that the large strain of the skin on the wrist reduced the tactile spatial acuity even though the small strain did not (Cody et al., 2010). Given that the reduced acuity is associated with exactly the opposite “shrunken” size (Von Békésy, 1975; Weber, 1984/1996), the influence of skin-based factors due to skin stretch may seem unlikely. Nevertheless, the skin tends to be less strained and more displaced in forearm rotation than it is when the wrist is bent. Thus, the influence of skin stretch remains a possibility to explore in future studies. The localization precision being improved at the medial-lateral axis (Cody et al., 2008) implicates the low-level factors related to somatotopy, and therefore the skin-based reference frame. These factors are further suggested by an overall distal bias in Experiment 2 (Azañón et al., 2010; Mancini et al., 2011; Margolis & Longo, 2015). However, the physical length of a *bent* forearm from wrist to elbow is longer than that from wrist to crease at the elbow on the inner side. This may have contributed to a reduced distal bias at the leftmost grid locations on the supinated forearm and rightmost grid locations on the pronated forearm, suggesting thus a possibility of skin-based factors being modulated by visual information of the limb length.

In discussing our experiments, we draw on evidence that the skin is represented in 2D somatosensory maps while it obviously wraps around the body of actual 3D shape given by the musculature and skeletal structure underneath the skin. It is straightforward to think about the experiments in terms of a displaced skin on the forearm’s surface, and the posture-induced rearrangement of the musculoskeletal structure underneath. It is plausible that the touch on skin surface and the musculoskeletal information provide partially incongruent information about spatial locations of sensory events in forearm torsion, and that these signals are integrated in a single estimation. However, it is important to note that it is not known if the influence of internal musculoskeletal factors is exerted by the *representation* of the body as a 3D construct, and it cannot be inferred with certainty from the evidence we present. Future research should determine if this integration may produce a 3D body representation by integrating, for example, the layers of two 2D maps, or if the outcome might still be a 2D representation that derives from the synthesis of 2D and/or 3D maps.

Final considerations should be given to differences across response modalities. The magnitude of the localization bias and the medial-lateral stretch were both smaller when the responses were given by pointing using the contralateral hand in Experiment 1. Additionally, the smaller response variability suggests that the task may have been easier when direct proprioceptive and motor feedback was allowed. The poorer performance for a dominantly visual response modality in Experiment 2 may thus be related to a more abstract nature of the task with an increased dependence on mental imagery. This suggests that the response modality, and thus higher-order factors played some role in the reported findings. For instance, the precision of localization was homogeneous across individual levels of manipulated variables in Experiment 1; however, it deteriorated for the supinated forearm relative to the pronated posture in Experiment 2. This finding may be attributed to the aforementioned allocentric view-angle for supinated forearm in the visual task which would not had been used by participants locating the touch by pointing to their own supinated forearm in Experiment 1. This interpretation is additionally consistent with the reduced advantage in localization precision at medial-lateral axis for supinated forearm in Experiment 2, that is, the reduction of the effect which is attributed to low-level somatosensory factors (Cody et al., 2008).

The strength of our pointing task are more direct measurements in actual body space. Its main weakness, which should be considered in future studies, may be in movement affordability. In comparison with Experiment 1, the task in Experiment 2 is unlikely to be equally sensitive. It is more abstract and therefore difficult, and it produces noisier data as the variable error results attest. Its strengths include (a) consistency in manual effort across all trials and (b) ease of access to all *perceived* tactile locations. It also helped us eliminate the ring of a large diameter used in Experiment 1 as a cause for the perceptual stretch of the grid. Further, this method allows dissociating of the pure effects of somatosensory and musculoskeletal factors from a feedback available from movements of the contralateral hand toward the stimulated region. Thus, both task have their strengths and shortcomings which should be weighed by researchers considering their use.

To conclude, we reported that the touch at *the same* skin location may be mislocalized in a different magnitude and direction as a function of forearm rotation. This finding adds to the existing body of research on tactile localization, by showing that the touch is not localized solely within a skin-based 2D reference frame and with respect to position of body part in the external space. It is additionally localized with reference to limb’s 3D make-up which may be spatially “rearranged” with the movement and changes in posture. Our study thus addressed an evident discontinuity in tactile localization research by focusing on the 3D body structure and implicating the use of musculoskeletal reference frame in localization of touch. At the same time, we developed paradigms to investigate the somatoperception (in this case tactile localization) while preserving the body’s three-dimensionality in response tasks.

## Supplementary Material

10.1037/xhp0000562.supp
